# Comparison of non-invasive serum markers for discriminating significant liver fibrosis in children with choledochal cyst

**DOI:** 10.3389/fped.2026.1992446

**Published:** 2026-10-27

**Authors:** Peng Zhang, Yaqin Zhen, Yuan Liu, Zheng Xu, Hongwei Xi

**Affiliations:** Department of General Surgery, Shanxi Provincial Children’s Hospital, Taiyuan, China

**Keywords:** children, choledochal cyst, gamma-glutamyl transpeptidase, liver fibrosis, non-invasive serum markers

## Abstract

**Objectives:**

To compare the discriminatory performance of seven serum markers for significant liver fibrosis in children with choledochal cyst (CDC) and to further explore the relationship between gamma-glutamyl transpeptidase (GGT) level and histologically significant liver fibrosis.

**Methods:**

We retrospectively collected clinical data of children with CDC who underwent radical surgery and intraoperative liver biopsy at Shanxi Provincial Children's Hospital from January 2020 to December 2025. Using METAVIR fibrosis stage as the histological reference standard, we compared seven serum markers: GGT, GGT to platelet ratio index (GPR), aspartate aminotransferase to alanine aminotransferase ratio (AAR), aspartate aminotransferase to platelet ratio index (APRI), Fibrosis-4 index (FIB-4), fibrosis cirrhosis index (FCI), and S-index. We evaluated the discriminatory performance of each marker using receiver operating characteristic (ROC) curves and analyzed the association between GGT and significant liver fibrosis using multivariate logistic regression.

**Results:**

We included 219 children, of whom 106 (48.4%) had significant liver fibrosis. GGT showed the highest correlation coefficient with METAVIR fibrosis stage (rs = 0.638, *P* < 0.001) and the highest AUC (0.826, 95% CI: 0.770–0.882), with an optimal cutoff of 123.0 U/L, sensitivity of 89.6%, and specificity of 69.9%. The AUCs for GPR, S-index, FCI, APRI, AAR, and FIB-4 were 0.814, 0.809, 0.802, 0.771, 0.705, and 0.528, respectively. After Holm correction, GGT showed no statistically significant difference in AUC compared with GPR, FCI, S-index, or APRI, and only outperformed AAR and FIB-4. After adjusting for age, sex, maximum cyst diameter, jaundice history, and cholangitis history, each 10 U/L increase in GGT was associated with an OR of 1.041 (95% CI: 1.021–1.062, *P* < 0.001) for significant liver fibrosis; after excluding children with a history of preoperative cholangitis, the OR was 1.055 (95% CI: 1.023–1.088, *P* < 0.001). The proportions of significant liver fibrosis in the GGT < 100, 100–300, and >300 U/L groups were 19.0%, 56.3%, and 69.6%, respectively, showing a linear increasing trend (*χ*^2^ trend = 42.90, *P* < 0.001). In the exploratory analysis for ≥F3, the AUCs for FCI and GGT were 0.906 and 0.901, respectively.

**Conclusions:**

In this cohort, GGT achieved the highest AUC but did not significantly outperform GPR, FCI, S-index, or APRI. GGT was independently associated with ≥F2 significant liver fibrosis, but this association may be partly influenced by biliary obstruction and cholangitis. GGT can serve as a serum marker to assist preoperative assessment of significant liver fibrosis, but it cannot replace histopathological evaluation.

## 1 Introduction

Choledochal cyst (CDC) is the most common congenital biliary malformation in children, characterized by cystic dilation of the intrahepatic or extrahepatic bile ducts (1). Cyst excision with Roux-en-Y hepaticojejunostomy has become the standard surgical procedure, but some children already have varying degrees of liver injury at the time of diagnosis. The core pathophysiology of CDC is cholestasis caused by biliary obstruction, and persistent obstruction can induce chronic liver injury and even liver fibrosis. Previous studies have shown that liver fibrosis is not uncommon in infants and young children with CDC, and some children may develop severe fibrosis or even cirrhosis (2, 3). Moreover, the degree of fibrosis is often more severe in infants than in older children (4). Although the degree of liver fibrosis usually does not change the basic indication for radical surgery or the standard surgical procedure in CDC, understanding the degree of liver fibrosis before surgery helps assess the extent of liver involvement and may provide useful information for perioperative management, parental counseling, and postoperative follow-up. However, liver biopsy, as the histological reference standard for fibrosis staging, has inherent limitations such as invasiveness, bleeding risk, and sampling error, making it difficult to use as a routine preoperative screening tool.

In recent years, non-invasive liver fibrosis models based on routine serum markers, such as aspartate aminotransferase-to-platelet ratio index (APRI) and Fibrosis-4 index (FIB-4), have been extensively validated in adults with chronic liver disease (5). However, these models mostly derive from adult chronic liver disease populations, and their variable composition was not designed specifically for pediatric CDC. Given that the core pathology of CDC is biliary obstruction and cholestasis, different pathophysiological backgrounds may lead to differences in the applicability of each serum marker in this population. Currently, validation of APRI and FIB-4 in pediatric CDC remains insufficient (6, 7), and systematic evaluation of cholestasis-specific markers and other novel composite models is still lacking. Therefore, direct comparative evidence based on a histological reference standard is lacking regarding the discriminatory performance of different serum markers in children with CDC. Because gamma-glutamyl transpeptidase (GGT) is an important indicator reflecting biliary injury and has potential disease relevance in the pathological process of pediatric CDC, we further analyzed its relationship with significant liver fibrosis. Based on this, the present study used intraoperative liver histopathology as the reference standard to compare the discriminatory performance of commonly used serum markers for significant liver fibrosis in children with CDC, and to analyze the association between GGT and significant liver fibrosis.

## 2 Materials and methods

### 2.1 Study population

This was a single-center retrospective study. We consecutively screened children diagnosed with CDC who underwent radical surgery at the Department of General Surgery, Shanxi Provincial Children's Hospital, between January 2020 and December 2025. During the study period, intraoperative liver biopsy was routinely performed in these children at our center, and cases were not selected based on the degree of liver fibrosis. Inclusion criteria were: (1) confirmed diagnosis of CDC by intraoperative exploration and/or cholangiography; (2) intraoperative liver biopsy with adequate pathological specimen; and (3) complete clinical data. Exclusion criteria were: (1) incomplete preoperative laboratory data; (2) inadequate pathological specimen; (3) coexisting other liver diseases; and (4) missing clinical data due to other reasons. This study was approved by the Ethics Committee of Shanxi Provincial Children's Hospital (IRB-KYYN-2022-004), and written informed consent was waived for the children's guardians.

### 2.2 Clinical data, laboratory indicators, and pathological evaluation

We collected the following data: sex, age (years), admission weight, maximum cyst diameter, Todani classification, preoperative abdominal pain, jaundice, and history of cholangitis, liver pathological fibrosis stage, and the most recent venous blood test results within 3 days before surgery, including alanine aminotransferase (ALT), aspartate aminotransferase (AST), GGT, albumin (ALB), total protein (Tp), total bilirubin (TBIL), direct bilirubin (DBIL), total bile acid (TBA), alkaline phosphatase (ALP), and platelet (PLT). Age was expressed in months for descriptive statistics and converted to years for logistic regression and FIB-4 calculation. According to the reagent kit instructions of our hospital's laboratory, the upper limit of normal (ULN) for GGT and AST was 40 U/L. Todani classification was determined based on preoperative imaging and intraoperative exploration findings.

Preoperative jaundice was based on medical records or clinical diagnosis. Preoperative cholangitis was determined based on clinical diagnosis in medical records, combined with fever, abdominal pain/right upper quadrant pain, jaundice, and infectious indicators. In this study, “history of preoperative cholangitis” referred to a diagnosis of cholangitis during the preoperative course. Maximum cyst diameter was obtained from the last preoperative imaging examination (abdominal ultrasound or magnetic resonance cholangiopancreatography); for multiple examinations, the one closest to the surgery date was used.

Liver pathology specimens were obtained intraoperatively from the anterior edge of the right hepatic lobe, all as wedge biopsies. Specimen adequacy required at least five portal tracts, and all pathological specimens meeting the inclusion criteria were evaluated according to this standard. Specimens were fixed in 10% neutral formalin, embedded in paraffin, and cut into 4-µm serial sections for hematoxylin-eosin staining and Masson's trichrome staining. Two pathologists independently performed liver fibrosis staging under a light microscope, both blinded to preoperative laboratory results and study group information. Disagreements were resolved through discussion to reach a consensus, with review by a senior pathologist when necessary. The METAVIR fibrosis staging system was used (8): F0, no fibrosis; F1, portal fibrosis without septa; F2, portal fibrosis with few septa; F3, numerous septa without cirrhosis; F4, cirrhosis. METAVIR has been used for fibrosis evaluation in some pediatric cholestatic liver disease and CDC-related studies.

### 2.3 Definitions and calculations of serum markers

Based on laboratory results within 3 days before surgery, we calculated the following serum markers: GGT to platelet ratio index (GPR), aspartate aminotransferase to alanine aminotransferase ratio (AAR), APRI, FIB-4, fibrosis cirrhosis index (FCI), and S-index. The formulas were as follows:

1. GGT: serum GGT raw value (U/L).
2. GPR = (GGT (U/L)/ULN of GGT) × 100/PLT (10^9^/L) (9).
3. AAR = AST (U/L)/ALT (U/L) (10).
4. APRI = (AST (U/L)/ULN of AST × 100)/PLT (10^9^/L) (11).
5. FIB-4 = [age (years) × AST (U/L)]/[PLT (10^9^/L) × √ALT (U/L)] (12).
6. FCI = [ALP (U/L) × TBIL (µmol/L)]/[ALB (g/L) × PLT (10^9^/L)] (13).
7. S-index = 1,000 × GGT (U/L)/[PLT (10^9^/L) × ALB^2^ (g/L)] (14).

### 2.4 Grouping and outcome definitions

1. Primary grouping: significant liver fibrosis

Patients were grouped according to the METAVIR fibrosis stage from intraoperative liver biopsy: F0, no fibrosis; F1, portal fibrosis without septa; F2, portal fibrosis with few septa; F3, numerous septa without cirrhosis; F4, cirrhosis (8). F0–F1 were classified as the no/mild fibrosis group, and F2–F4 as the significant fibrosis group (≥F2). ≥F2 was used as the pathological threshold for histologically significant liver fibrosis and as the primary endpoint for evaluating discriminatory performance. F2 and above are commonly used as the threshold for significant fibrosis in liver fibrosis studies, and previous pediatric cholestatic liver disease studies also used this binary classification (15).

2. Exploratory outcome: advanced fibrosis

To evaluate the ability of serum markers to identify more severe fibrosis, we further defined F3–F4 as advanced fibrosis, with F0–F2 as the control group for exploratory analysis.

3. Stratified analysis by GGT level

To further explore the relationship between GGT level and the proportion of significant liver fibrosis, we used 100 U/L and 300 U/L as exploratory stratification cutoffs and divided children into three groups: GGT < 100 U/L, 100 ≤ GGT ≤ 300 U/L, and GGT > 300 U/L. Because previous study thresholds have not been unified, this study also used an exploratory stratification method to evaluate changes in fibrosis proportion across different GGT levels. The basis for the stratification cutoffs was as follows: (1) 100 U/L: in previous studies on infantile cholestatic diseases, GGT ≤ 100 U/L was considered a cutoff for low/normal GGT status (16); moreover, infant GGT levels show clear age dependence, with considerable variation in reference ranges across different age groups (17). (2) 300 U/L: a relevant pediatric CDC study reported that the optimal GGT cutoff for diagnosing significant liver fibrosis was 327 U/L (18); another study on children with congenital biliary dilatation observed that those with fibrosis beyond F1 often had persistent GGT > 300 U/L (19).

### 2.5 Statistical analysis

We performed statistical analysis using SPSS 26.0. The Shapiro–Wilk test combined with *Q*–*Q* plots was used to assess the distribution of continuous variables. Normally distributed continuous data were expressed as mean ± standard deviation and compared between groups using the independent-samples *t*-test; non-normally distributed continuous data were expressed as median (P25, P75) and compared using the Mann–Whitney *U* test. Categorical data were expressed as counts (percentages) and compared using the *χ*^2^ test, or Fisher's exact test when expected frequencies were <5. The 95% CI for proportions was calculated using the Clopper–Pearson exact binomial method. Missing data for variables included in the analysis were described. Spearman's rank correlation test was used to evaluate the correlation between each serum marker and METAVIR fibrosis stage. ROC curves were plotted for the primary outcome of METAVIR ≥ F2, and the area under the curve (AUC) and 95% CI (DeLong method) were calculated. The optimal cutoff was determined using the Youden index, along with the corresponding sensitivity, specificity, positive predictive value (PPV), and negative predictive value (NPV). AAR was considered positive when below or equal to the cutoff, while other markers were considered positive when above or equal to the cutoff. For markers with the opposite direction, the positive classification direction was adjusted according to the ROC curve direction before calculating the Youden index. The DeLong test was used to compare AUCs among markers, and the Holm method was applied to correct for multiple comparisons in pairwise comparisons. Multivariate logistic regression was performed with the presence or absence of METAVIR ≥ F2 as the dependent variable. The primary model included age, sex, maximum cyst diameter, preoperative jaundice history, and preoperative cholangitis history. The extended model added TBIL, ALP, and TBA to the primary model and was mainly used for exploratory analysis. Continuous GGT was analyzed per 10 U/L increase, and results were expressed as OR and 95% CI. Variance inflation factor (VIF) was used to evaluate collinearity among model variables. Considering age and sex differences in pediatric AST and GGT reference ranges, sensitivity analyses were performed: GGT and GPR were re-standardized using age/sex-appropriate upper limits of normal, APRI was re-analyzed after excluding children aged <1 year, and FIB-4 was calculated using the standard formula with age in years. A 2,000-iteration bootstrap resampling was used to evaluate the uncertainty of AUC and optimal cutoff, with the distribution range of the optimal cutoff during resampling expressed as the 2.5th–97.5th percentile. The trend between GGT level and the proportion of ≥F2 significant liver fibrosis was tested using the *χ*^2^ trend test. A two-sided *P* < 0.05 was considered statistically significant.

## 3 Results

### 3.1 Patient inclusion and baseline characteristics

During the study period, we consecutively screened 245 children with CDC who underwent radical surgery. Of these, 26 were excluded, and 219 were finally included. Reasons for exclusion were: incomplete preoperative laboratory data in 12 cases, inadequate pathological specimens in 5 cases, coexisting other liver diseases in 3 cases, perioperative death with incomplete data in 1 case, and other reasons in 5 cases. Demographic characteristics, clinical features, and distribution of liver fibrosis stages of the 219 children are shown in Table 1. Among the 219 children, 54 (24.7%) were male and 165 (75.3%) were female, with a median age of 47.0 months, median admission weight of 15.4 kg, and median maximum cyst diameter of 27.0 mm. Todani classification was predominantly type Ia and type IVa, accounting for 121 cases (55.3%) and 85 cases (38.8%), respectively. The remaining 13 cases (5.9%) included type IVb in 7 cases (3.2%), type Ib in 3 cases (1.4%), type Ic in 2 cases (0.9%), and type II in 1 case (0.5%). Preoperative abdominal pain was present in 168 children (76.7%), jaundice in 51 (23.3%), and cholangitis in 120 (54.8%). The distribution of METAVIR liver fibrosis stages (Figure 1) was as follows: F0 in 10 cases (4.6%), F1 in 103 cases (47.0%), F2 in 74 cases (33.8%), F3 in 25 cases (11.4%), and F4 in 7 cases (3.2%). No missing values were found for major laboratory indicators in the included cases.

**Table 1:** Demographic characteristics and distribution of liver fibrosis stages in children with choledochal cyst.

Characteristic	Value
Sex (male/female) [*n* (%)]	54/165 (24.7%/75.3%)
Age (months)	47.0 (26.0, 84.0)
Admission weight (kg)	15.4 (12.0, 22.5)
Maximum cyst diameter (mm)	27.0 (17.0, 46.5)
Todani classification [*n* (%)]	
Type Ia	121 (55.3%)
Type IVa	85 (38.8%)
Type IVb	7 (3.2%)
Type Ib	3 (1.4%)
Type Ic	2 (0.9%)
Type II	1 (0.5%)
Preoperative abdominal pain	168 (76.7%)
Preoperative jaundice	51 (23.3%)
Preoperative cholangitis	120 (54.8%)
Liver fibrosis stage [*n* (%)]	
F0	10 (4.6%)
F1	103 (47.0%)
F2	74 (33.8%)
F3	25 (11.4%)
F4	7 (3.2%)

**Figure 1:**
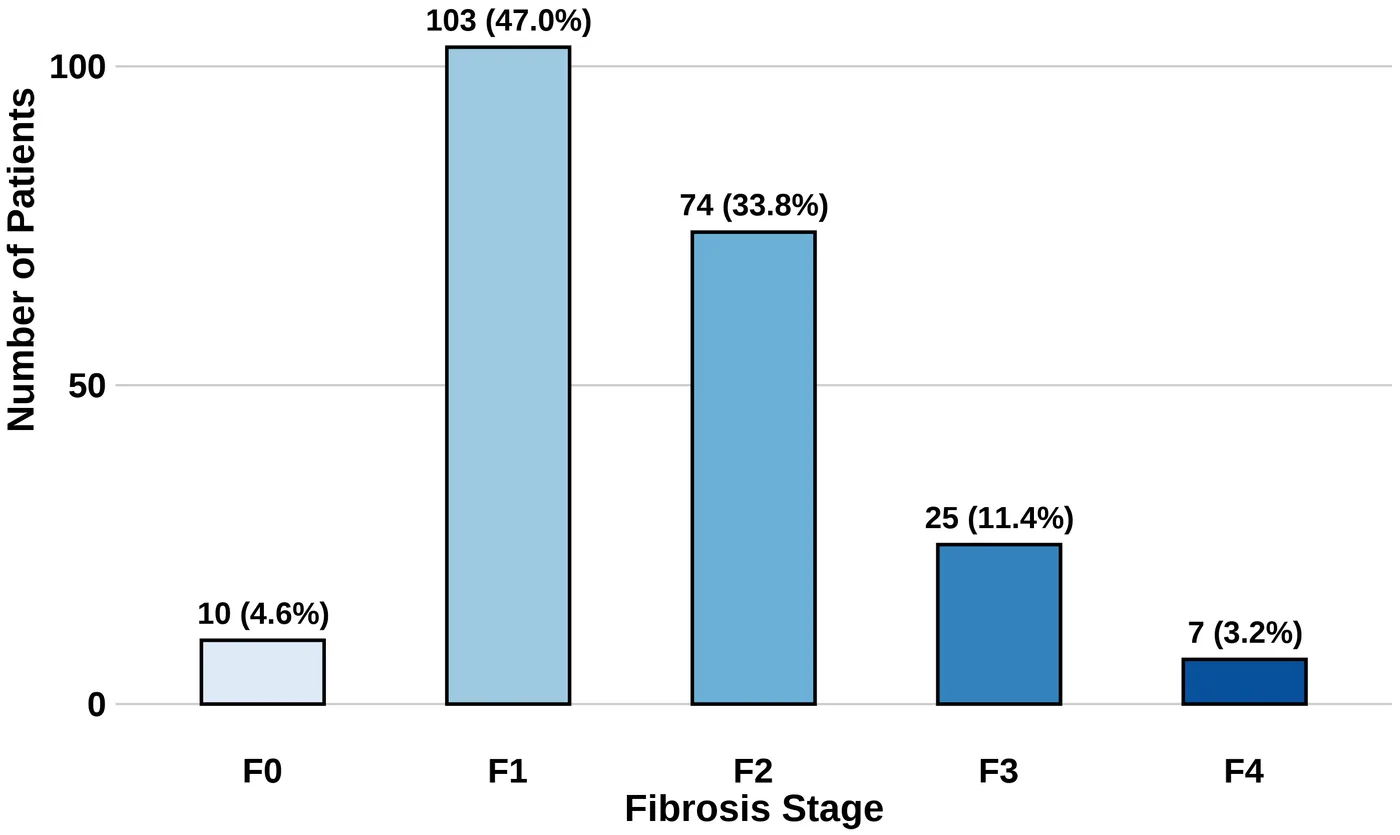
Distribution of METAVIR liver fibrosis stages in children with choledochal cyst.

### 3.2 Comparison of baseline characteristics between fibrosis groups

Based on METAVIR fibrosis stage, we divided the 219 children into the no/mild fibrosis group (F0–F1, *n* = 113) and the significant fibrosis group (≥F2, *n* = 106). Baseline characteristics of the two groups are compared in Table 2. There were no statistically significant differences in age, admission weight, or preoperative abdominal pain history between the two groups. The significant fibrosis group had a higher proportion of females, a larger maximum cyst diameter, and significantly higher rates of preoperative jaundice and cholangitis history than the no/mild fibrosis group. Regarding laboratory indicators, the significant fibrosis group had significantly higher levels of GGT, ALT, AST, ALP, TBIL, DBIL, TBA, and PLT than the no/mild fibrosis group, while ALB and Tp showed no significant differences between the two groups. For serum non-invasive models, GPR, APRI, FCI, and S-index were significantly higher in the significant fibrosis group; AAR was lower in the significant fibrosis group than in the no/mild fibrosis group; and FIB-4 showed no significant difference between the two groups.

**Table 2:** Comparison of baseline characteristics and serum marker models between the no/mild fibrosis group and the significant fibrosis group.

Variable	No/mild fibrosis group (F0–F1, *n* = 113)	Significant fibrosis group (≥F2, *n* = 106)	Statistic value (*Z*/*χ*^2^)	*P* value
Sex			*χ*^2^ = 6.516	0.011
Male	36	18		
Female	77	88		
Age (months)	50.0 (26.0, 84.0)	40.5 (26.0, 84.5)	*Z* = −0.688	0.491
Admission weight (kg)	16.0 (12.2, 22.4)	15.1 (11.9, 22.8)	*Z* = −0.664	0.507
Maximum cyst diameter (mm)	23.0 (17.0, 38.0)	32.5 (17.0, 49.3)	*Z* = 2.186	0.029
Preoperative abdominal pain [*n* (%)]	82 (72.6)	86 (81.1)	*χ*^2^ = 2.246	0.134
Preoperative jaundice [*n* (%)]	17 (15.0)	34 (32.1)	*χ*^2^ = 8.881	0.003
Preoperative cholangitis [*n* (%)]	38 (33.6)	82 (77.4)	*χ*^2^ = 42.225	<0.001
Laboratory markers				
ALT (U/L)	23.0 (13.5, 100.0)	207.7 (107.8, 333.0)	*Z* = 7.893	<0.001
AST (U/L)	37.0 (26.1, 74.1)	149.8 (77.3, 265.1)	*Z* = 7.644	<0.001
GGT (U/L)	58.0 (15.0, 187.0)	356.5 (184.0, 573.0)	*Z* = 8.336	<0.001
Tp (g/L)	65.7 (62.1, 69.0)	65.7 (62.0, 69.2)	*Z* = −0.041	0.968
ALB (g/L)	44.4 (41.2, 46.7)	43.9 (41.2, 45.8)	*Z* = −0.962	0.336
TBIL (μmol/L)	9.9 (7.0, 16.3)	30.4 (14.3, 65.2)	*Z* = 7.406	<0.001
DBIL (μmol/L)	3.6 (2.5, 5.8)	21.9 (6.2, 55.4)	*Z* = 7.773	<0.001
TBA (μmol/L)	3.0 (1.4, 6.7)	14.7 (3.6, 75.0)	*Z* = 5.625	<0.001
ALP (U/L)	228.0 (183.0, 294.0)	347.5 (275.0, 455.0)	*Z* = 7.131	<0.001
PLT (×10^9^/L)	319.0 (277.0, 391.0)	374.0 (289.0, 430.0)	*Z* = 2.422	0.015
Serum non-invasive models				
GPR	0.359 (0.119, 1.388)	2.701 (1.335, 4.094)	*Z* = 8.032	<0.001
AAR	1.504 (0.789, 2.125)	0.838 (0.586, 1.326)	*Z* = −5.236	<0.001
APRI	0.287 (0.197, 0.537)	0.955 (0.498, 1.957)	*Z* = 6.926	<0.001
FIB-4	1.175 (0.585, 2.362)	1.427 (0.636, 2.254)	*Z* = 0.715	0.475
FCI	0.156 (0.095, 0.312)	0.818 (0.281, 1.923)	*Z* = 7.728	<0.001
S-index	0.072 (0.024, 0.310)	0.539 (0.266, 0.916)	*Z* = 7.884	<0.001

### 3.3 Correlation between seven serum markers and METAVIR stage

Spearman rank correlation analysis (Table 3, Figure 2) showed considerable variation in the correlations between the seven serum markers and METAVIR fibrosis grade. GGT, GPR, APRI, FCI, and S-index all showed significant positive correlations with fibrosis grade, with GGT having the highest correlation coefficient (rs = 0.638, *P* < 0.001). AAR showed a significant negative correlation with fibrosis stage (rs = −0.349, *P* < 0.001). FIB-4 showed no statistically significant correlation with METAVIR fibrosis stage (rs = 0.093, *P* = 0.170). Regarding inter-marker correlations, GPR and S-index, GGT and GPR, and GGT and S-index showed strong correlations. In addition, GPR and APRI, as well as GGT and FCI, showed significant correlations. FIB-4 showed no statistically significant correlation with GGT, GPR, or S-index, but showed low-degree correlations with AAR, APRI, and FCI (rs = 0.176, *P* = 0.009; rs = 0.344, *P* < 0.001; rs = 0.212, *P* = 0.002, respectively). GPR, S-index, and GGT showed strong correlations with each other (rs = 0.985, 0.972, and 0.963, respectively).

**Table 3:** Correlation between seven serum markers and METAVIR fibrosis grade.

Marker	*r*_s_ value	*P* value
GGT	0.638	<0.001
GPR	0.608	<0.001
AAR	−0.349	<0.001
APRI	0.533	<0.001
FIB-4	0.093	0.170
FCI	0.603	<0.001
S-index	0.608	<0.001

**Figure 2:**
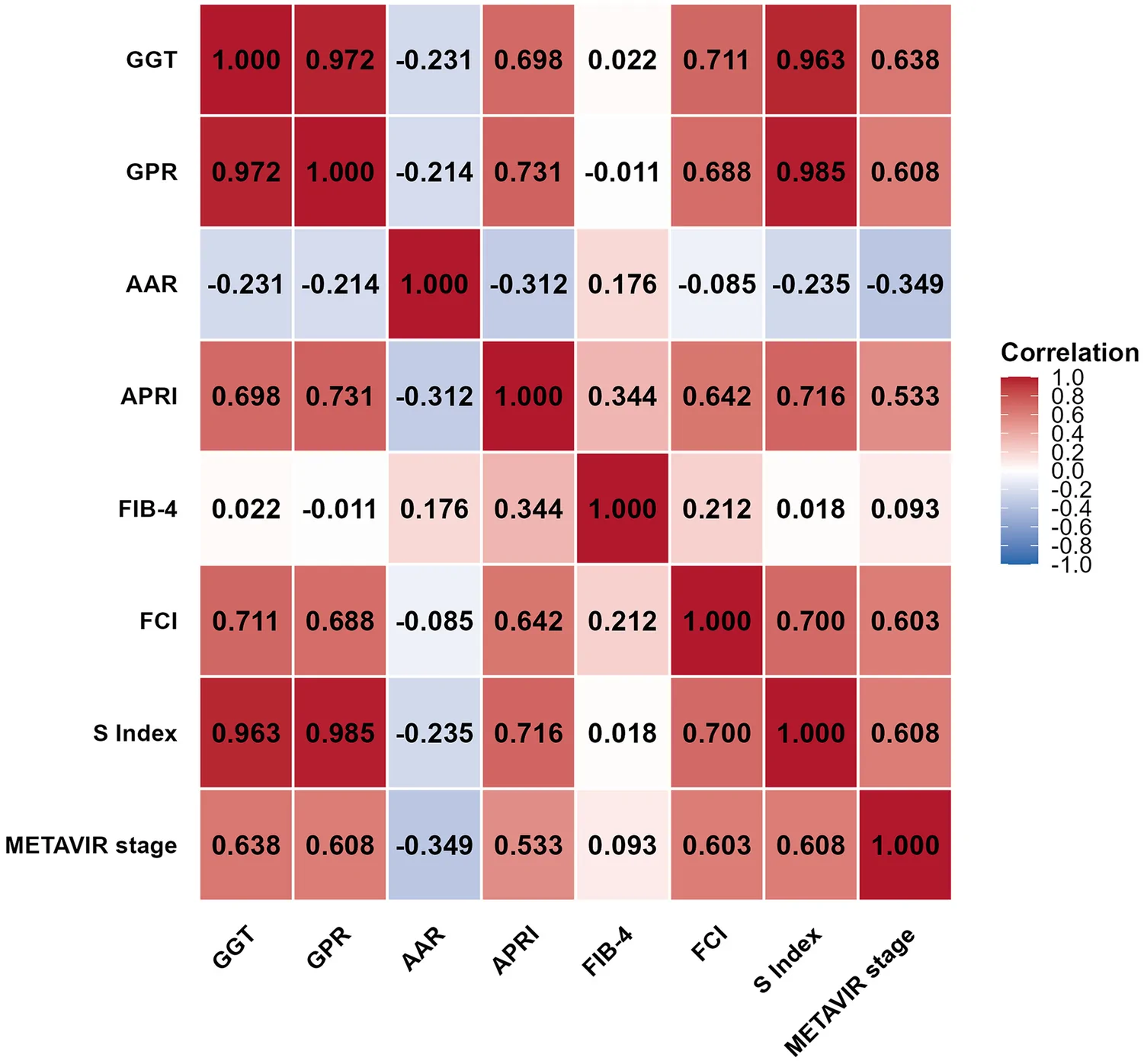
Correlation heatmap between serum markers and METAVIR liver fibrosis stage.

### 3.4 ROC curve analysis of seven serum markers for diagnosing significant liver fibrosis

Using METAVIR ≥ F2 as the primary endpoint, we performed ROC analysis for the seven serum markers (Table 4, Figure 3). GGT, GPR, S-index, and FCI all had AUC values exceeding 0.80, with GGT having the highest numerical AUC of 0.826 (95% CI: 0.770–0.882); the AUCs for APRI, AAR, and FIB-4 were 0.771, 0.705, and 0.528, respectively. The optimal cutoff for GGT in this cohort based on the Youden index was 123.0 U/L, with a sensitivity of 89.6% and specificity of 69.9%. After Holm correction, the DeLong test showed no statistically significant differences in AUC between GGT and GPR, FCI, or S-index; the difference between GGT and APRI was statistically significant before correction (*P* = 0.039) but not after correction (*P* = 0.430); the differences between GGT and AAR and FIB-4 remained statistically significant after correction (*P* = 0.002 and *P* < 0.001, respectively). The 2 × 2 classification counts corresponding to the optimal cutoff for each marker are shown in Supplementary Table S1, and the complete results of the 21 pairwise comparisons are shown in Supplementary Table S2.

**Table 4:** Comparison of diagnostic performance of each serum marker model for significant liver fibrosis (≥F2).

Marker	AUC (95% CI)	Optimal cutoff	Direction	Sensitivity	Specificity	PPV	NPV	Youden index
GGT	0.826 (0.770–0.882)	123.0	≥	89.6%	69.9%	73.6%	87.8%	0.595
GPR	0.814 (0.756–0.872)	0.857	≥	87.7%	69.9%	73.2%	85.9%	0.576
AAR	0.705 (0.636–0.774)	1.141	≤	71.7%	63.7%	65.0%	70.6%	0.354
APRI	0.771 (0.707–0.835)	0.406	≥	83.0%	70.8%	72.7%	81.6%	0.538
FIB-4	0.528 (0.451–0.605)	Not applicable	Not applicable	59.4%	53.1%	54.3%	58.3%	0.125
FCI	0.802 (0.744–0.861)	0.230	≥	84.9%	68.1%	71.4%	82.8%	0.530
S-index	0.809 (0.750–0.867)	0.152	≥	89.6%	67.3%	72.0%	87.4%	0.569

Direction refers to the cutoff direction for defining a positive result; AAR uses ≤ cutoff, and the other markers use ≥ cutoff. The AUC of FIB-4 was close to the random level (0.528), and its optimal cutoff based on the Youden index has no clinical interpretability; therefore, the cutoff and direction are not listed.

**Figure 3:**
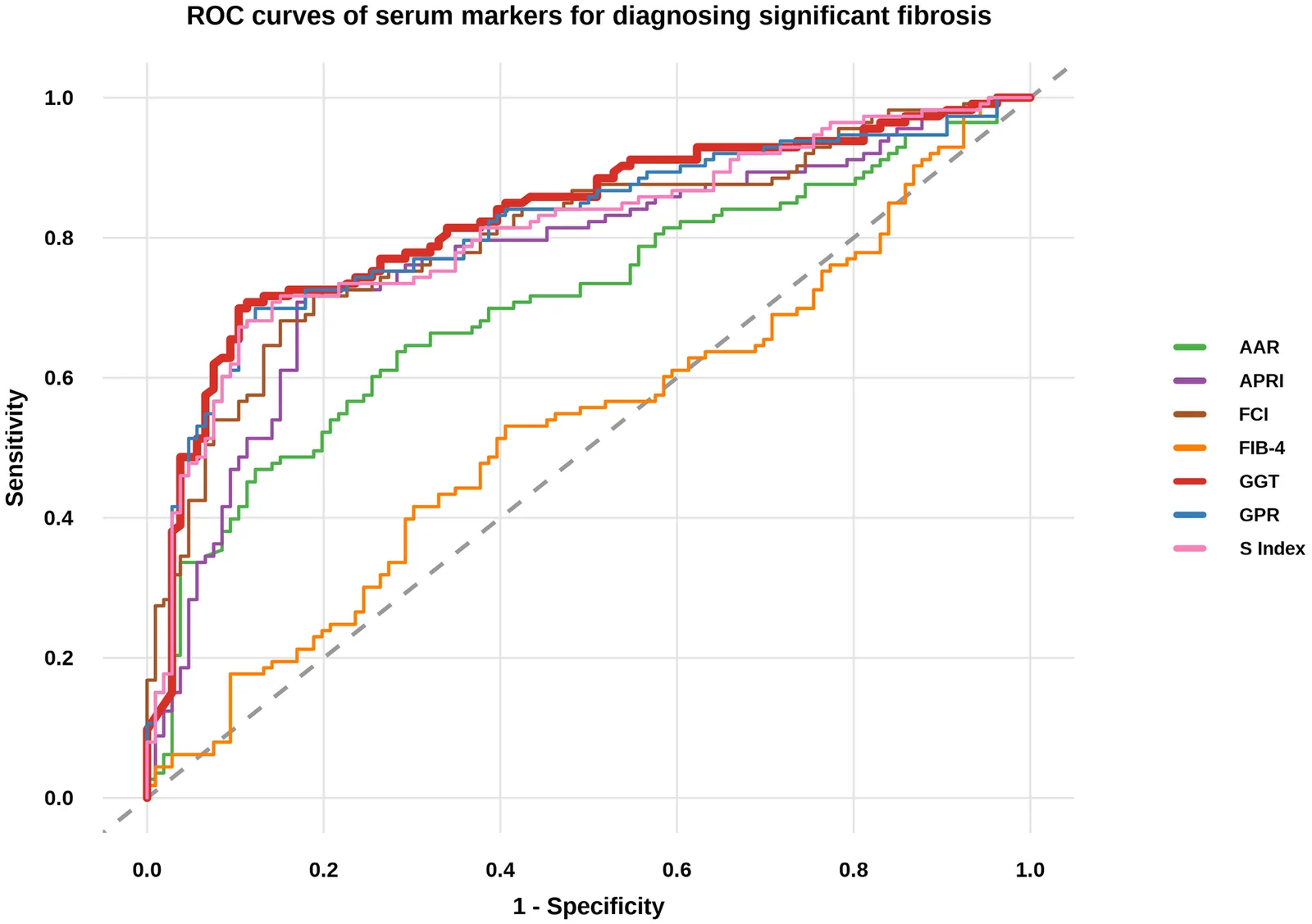
ROC curves of each serum marker model for diagnosing significant liver fibrosis (≥F2) in children with choledochal cyst.

### 3.5 Adjusted association between GGT and significant liver fibrosis and sensitivity analysis

We performed multivariate logistic regression analysis with METAVIR ≥ F2 as the dependent variable. The primary model included age, sex, maximum cyst diameter, preoperative jaundice history, and preoperative cholangitis history; the extended model added TBIL, ALP, and TBA to the primary model. Results showed that in the primary model, each 10 U/L increase in GGT was associated with an OR of 1.041 (*P* < 0.001); in the extended model, each 10 U/L increase in GGT was associated with an OR of 1.036 (*P* < 0.001). Preoperative cholangitis history (OR = 4.297, *P* < 0.001) and maximum cyst diameter (OR = 1.017, *P* = 0.039) were also associated with ≥F2 significant liver fibrosis, while other variables showed no statistically significant differences (Table 5). All included variables had VIF < 5. After excluding all children with a history of preoperative cholangitis, 99 cases remained; the AUC of GGT for discriminating ≥F2 significant liver fibrosis was 0.771; after adjusting for age, sex, maximum cyst diameter, and jaundice history, GGT remained statistically associated with ≥F2 significant liver fibrosis (OR = 1.055 per 10 U/L increase, *P* < 0.001). Age/sex-appropriate ULN sensitivity analysis showed that the AUC of GPR after correction was 0.808 (original: 0.814), the AUC of APRI after excluding 14 children aged <1 year was 0.766 (original: 0.771), and the AUC of GGT after standardization by age/sex ULN was 0.821 (original: 0.826). Bootstrap resampling stability analysis showed that the interval estimates of AUC for each marker were generally consistent with the primary analysis, and the resampling distribution range of the optimal cutoff for GGT was 86–161 U/L. The optimal cutoff of FIB-4 showed poor stability in Bootstrap analysis. The detailed results are shown in Supplementary Table S3.

**Table 5:** Multivariate logistic regression analysis of factors associated with significant liver fibrosis (≥F2).

Variable	Primary model OR (95% CI)	Extended model OR (95% CI)
GGT (per 10 U/L increase)	1.041 (1.021–1.062)^a^	1.036 (1.016–1.057)^a^
Age (per 1-year increase)	1.029 (0.937–1.130)	1.029 (0.937–1.130)
Female	1.997 (0.913–4.369)	1.997 (0.913–4.369)
Maximum cyst diameter (per 1-mm increase)	1.017 (1.001–1.034)^b^	1.017 (1.001–1.034)^b^
Jaundice history	1.157 (0.388–3.449)	1.157 (0.388–3.449)
Cholangitis history	4.297 (2.104–8.778)^a^	4.297 (2.104–8.778)^a^
TBIL	—	0.998 (0.986–1.011)
ALP	—	1.001 (0.998–1.004)
TBA	—	0.998 (0.993–1.003)

The primary model included age, sex, maximum cyst diameter, jaundice history, and cholangitis history; the extended model added TBIL, ALP, and TBA to the primary model.

a *P* < 0.001.

b *P* < 0.05.

### 3.6 GGT level stratification and proportion of significant liver fibrosis

To further examine the relationship between GGT level and the proportion of significant liver fibrosis, we used 100 U/L and 300 U/L as exploratory stratification cutoffs and divided the children into three groups. Results showed that the proportions of significant liver fibrosis (≥F2) in the GGT < 100 U/L, 100 ≤ GGT ≤ 300 U/L, and >300 U/L groups were 19.0%, 56.3%, and 69.6%, respectively, showing a significant linear increasing trend (*χ*^2^ trend = 42.90, *P* < 0.001) (Table 6, Figure 4).

**Table 6:** Proportion of significant liver fibrosis (≥F2) across different GGT levels.

GGT group	Total cases (*n*)	Cases with ≥F2 (n)	Prevalence of significant fibrosis (%)	95% CI (%)
<100 U/L	79	15	19.0	11.0–29.0
100–300 U/L	48	27	56.3	41.2–70.5
>300 U/L	92	64	69.6	59.1–78.7

*χ*^2^ trend test = 42.90, *P* < 0.001, indicating a significant linear increasing trend between GGT level and the proportion of significant fibrosis. The 95% CI for proportions was calculated using the Clopper-Pearson exact binomial method.

**Figure 4:**
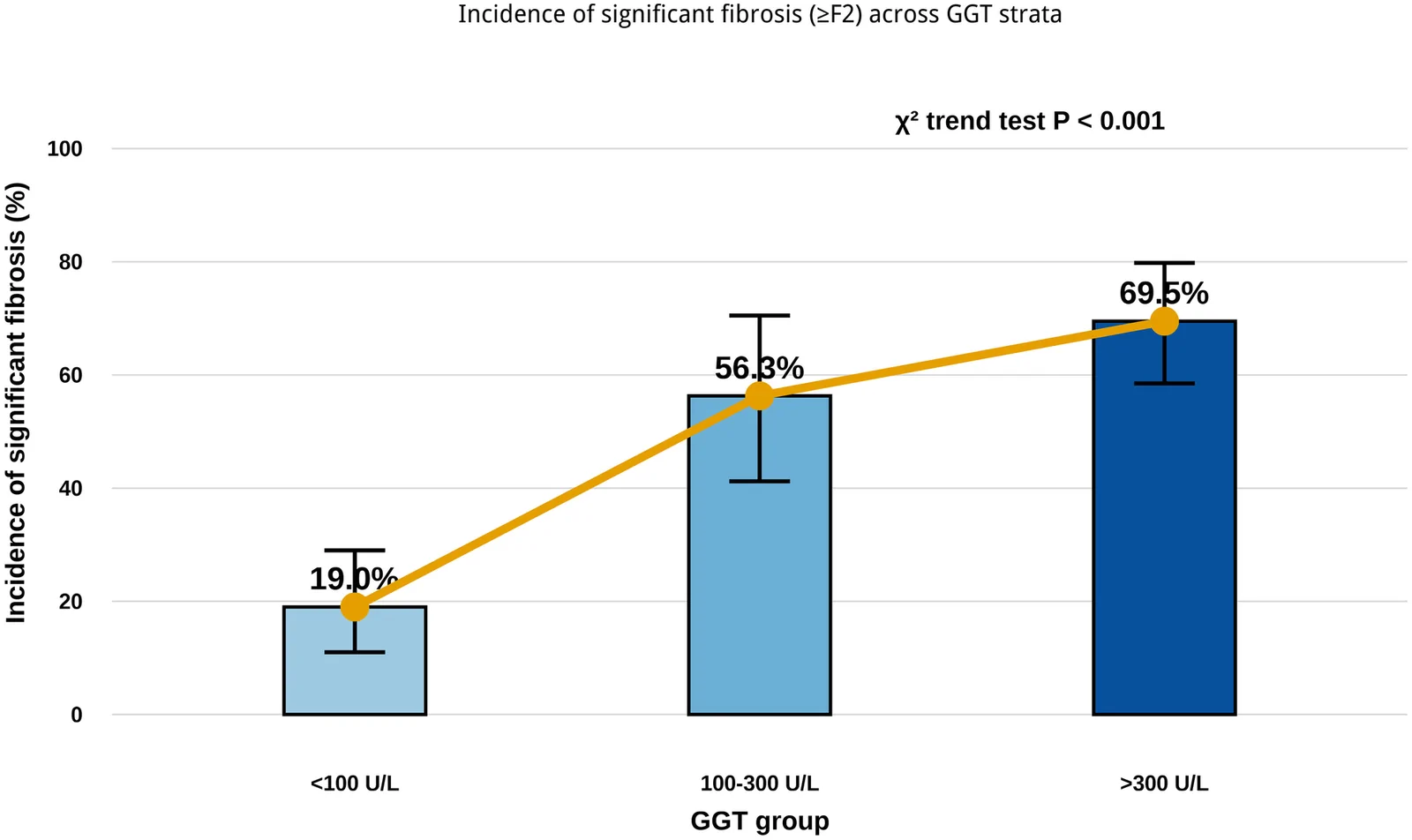
Proportion and trend of significant liver fibrosis (≥F2) across different GGT levels.

### 3.7 Exploratory ROC analysis of seven serum markers for diagnosing advanced fibrosis (≥F3)

Using advanced fibrosis (≥F3) as the positive event (32 cases, 14.6%), we performed exploratory ROC curve analysis for the seven serum markers (Table 7, Figure 5). Among the markers, FCI had the highest AUC (0.906), followed by GGT (0.901); the AUCs for AAR and FIB-4 were 0.587 and 0.567, respectively.

**Table 7:** Comparison of diagnostic performance of each serum marker model for advanced liver fibrosis (≥F3) (exploratory analysis).

Marker	AUC (95% CI)	Optimal cutoff	Direction	Sensitivity	Specificity	PPV	NPV
FCI	0.906 (0.860–0.951)	0.469	≥	96.9%	69.5%	35.2%	99.2%
GGT	0.901 (0.854–0.948)	342	≥	90.6%	77.5%	40.8%	97.9%
S-index	0.884 (0.836–0.933)	0.533	≥	90.6%	75.9%	39.2%	97.9%
GPR	0.868 (0.816–0.920)	1.893	≥	93.8%	67.4%	33.0%	98.4%
APRI	0.846 (0.791–0.900)	0.711	≥	93.8%	67.9%	33.3%	98.4%
AAR	0.587 (0.504–0.670)	1.271	≤	84.4%	48.1%	21.8%	94.7%
FIB-4	0.567 (0.483–0.651)	0.1085	≥	65.6%	51.3%	18.8%	89.7%

This analysis was exploratory. Because only 32 positive cases were included, the cutoff values and diagnostic performance need to be validated in larger samples.

**Figure 5:**
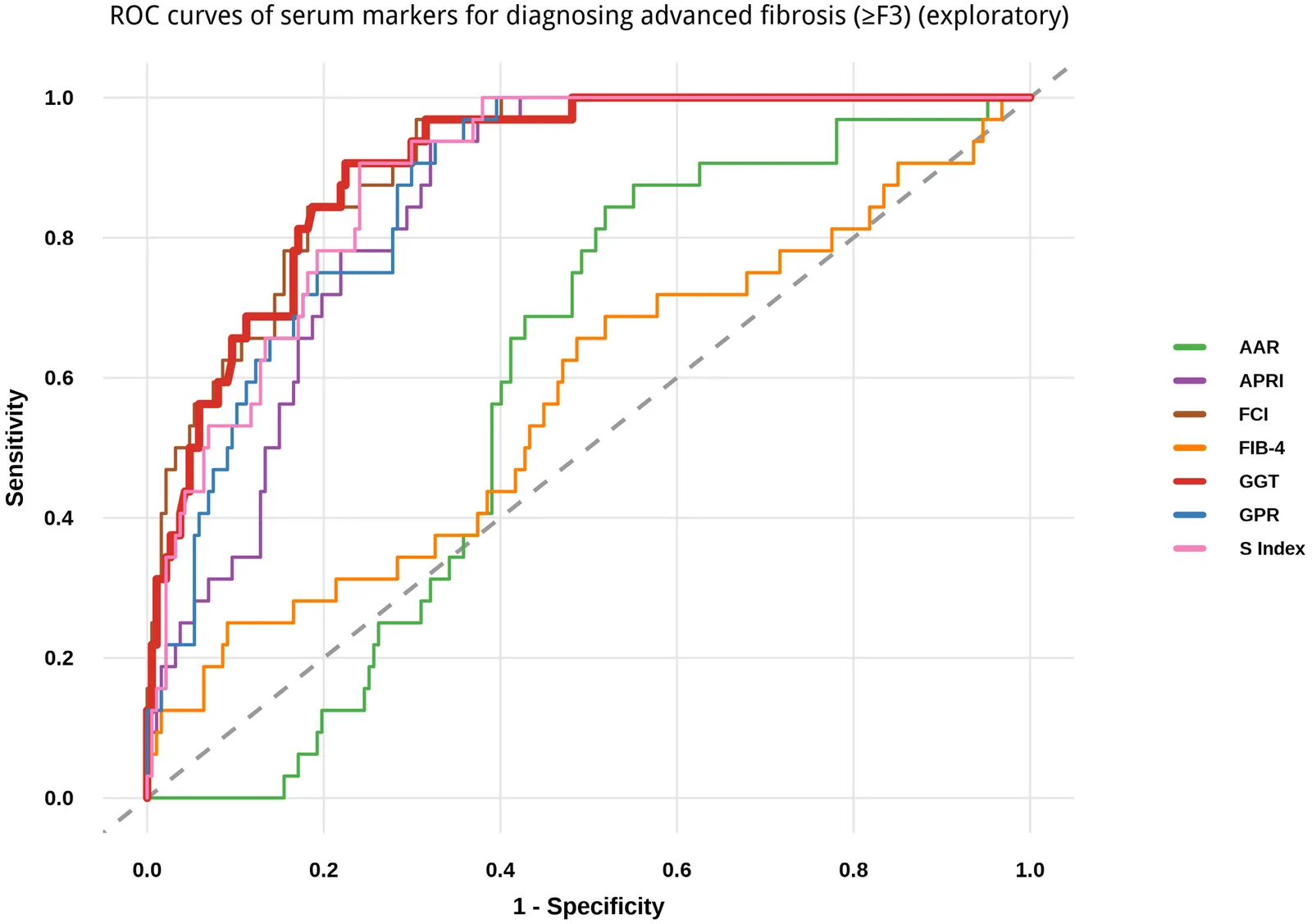
ROC curves of each serum marker model for diagnosing advanced liver fibrosis (≥F3) (exploratory analysis).

## 4 Discussion

This study included 219 children with CDC who underwent surgery and intraoperative liver biopsy, and compared the discriminatory performance of seven routine serum markers for histologically significant liver fibrosis (≥F2). GGT showed the highest correlation coefficient with METAVIR fibrosis stage (rs = 0.638) and the highest AUC (0.826); however, after Holm correction, the AUC differences between GGT and GPR, FCI, S-index, or APRI were not statistically significant. Therefore, our results suggest that GGT has relatively good discriminatory performance, but this is insufficient to conclude that it is superior to the other markers with similar performance. Multivariate analysis showed that after adjusting for age, sex, maximum cyst diameter, jaundice history, and cholangitis history, GGT remained statistically associated with ≥F2 significant liver fibrosis; after further adjusting for TBIL, ALP, and TBA, the association persisted, with little change in the effect size of GGT.

The relatively good performance of GGT in this study may be related to its position in the pathological process of CDC. CDC is characterized by biliary obstruction and cholestasis, and GGT is more directly affected by cholestasis and bile duct injury. In contrast, APRI and FIB-4 are mainly based on AST, ALT, and PLT, and in diseases dominated by biliary obstruction, the pathological information reflected by these markers may differ from that of GGT. Chen et al. (6) reported in a cohort of 330 children with CDC that the AUC of APRI for diagnosing advanced fibrosis was 0.761, while that of FIB-4 was only 0.561. Although the study endpoint was not completely consistent with ours, it similarly reflects the limitations of adult-derived models in pediatric CDC. Zhuang et al. (18) also reported that GGT is associated with significant liver fibrosis in CDC. These results suggest that when evaluating serum markers in pediatric CDC, the disease characteristics of biliary obstruction and cholestasis should be considered, and the performance of adult chronic liver disease models should not be directly extrapolated to children with CDC.

In this study, the incidence of preoperative cholangitis history in the ≥F2 group was 77.4%, significantly higher than 33.6% in the F0–F1 group, and TBIL, DBIL, ALP, and TBA were also elevated, suggesting that the association between GGT and fibrosis may partly reflect the coexisting biliary obstruction and inflammatory state. Therefore, we further performed multivariate analysis. After adjusting for age, sex, maximum cyst diameter, jaundice history, cholangitis history, TBIL, ALP, and TBA, GGT remained statistically associated with ≥F2 significant liver fibrosis; after excluding all children with a history of preoperative cholangitis, the association persisted (OR = 1.055, 95% CI: 1.023–1.088, *P* < 0.001), but the AUC decreased from 0.826 to 0.771, indicating that its discriminatory ability may be partly influenced by the biliary inflammatory background. It should be noted that GGT, TBIL, ALP, and TBA all belong to the cholestasis/biliary injur*y* axis. Although VIF < 5 suggests no severe collinearity, overadjustment and shared pathological processes cannot be completely excluded. The purpose of constructing the extended model was to observe whether the association between GGT and significant liver fibrosis is independent of some cholestasis-related indicators. However, given that these indicators may lie in the same pathway of CDC biliary obstruction–cholestasis–liver injury, and the extended model has a limited number of variables, this model should be regarded as exploratory analysis only, and should not be used to infer the effect of GGT independent of cholestatic factors. Because TBIL and DBIL showed obvious collinearity, the final model used TBIL to represent bilirubin level. The results of this model should be interpreted as conditional associations after adjusting for prespecified covariates, rather than establishing an independent prediction model or inferring causality. In addition, maximum cyst diameter was also weakly associated with significant liver fibrosis (OR = 1.017, 95% CI: 1.001–1.034, *P* = 0.039). Cyst size may reflect long-term biliary dilation and cholestasis, which has some biological plausibility, but the effect size is small and still needs further validation in combination with disease duration and dynamic imaging data.

GPR and S-index incorporate PLT and ALB, respectively, into the GGT framework, and theoretically can simultaneously reflect biliary injury, portal hypertension, and hepatic synthetic function. In this study, the AUCs of GPR, S-index, and FCI all exceeded 0.80, but the differences from GGT were not statistically significant; GGT was only superior to AAR and FIB-4 after correction. Correlation analysis showed that GPR and S-index (rs = 0.985), GGT and GPR (rs = 0.972), and GGT and S-index (rs = 0.963) were highly correlated, possibly reflecting highly consistent information due to shared variables. In this cohort, no statistically significant AUC gain was observed for GPR or S-index relative to GGT. In addition, PLT in the significant fibrosis group was higher than in the no/mild fibrosis group (*P* = 0.015), which differs from the thrombocytopenia pattern commonly seen in adult advanced cirrhosis and may be related to the inflammatory state and disease spectrum in pediatric biliary diseases; however, due to the lack of data on portal pressure and spleen, further explanation is not possible.

FIB-4 showed low discriminatory ability in this study (AUC = 0.528). FIB-4 was originally established in an adult chronic liver disease population (12), and its combination of age, AST, ALT, and PLT was not designed for pediatric CDC. Pediatric AST and ALT reference ranges vary significantly with age, and the main mechanism of liver injury in CDC is biliary obstruction, cholestasis, and cholangitis. Therefore, directly applying adult-derived FIB-4 may have insufficient applicability. This study used the standard FIB-4 formula with age in years, so its low discriminatory ability is not due to unit adjustment of the formula. The AUC of APRI was 0.771, which was higher than FIB-4 but still lower than GGT; AAR showed an inverse change, possibly related to the fact that ALT elevation was significantly greater than AST elevation in the ≥F2 group. These results suggest that when evaluating non-invasive serum markers in pediatric CDC, both age-related characteristics of children and the pathophysiological background of the disease should be considered. FCI includes ALP, and ALP has age dependence in childhood; although this study did not observe a significant correlation between FCI and age (rs = −0.044, *P* = 0.512), the influence of age-related physiological ALP changes on the discriminatory performance of FCI cannot be completely excluded. In the exploratory ≥F3 analysis, FCI showed a relatively high AUC (0.906), but this analysis included only 32 positive cases and was used only to explore the potential ability of different markers to identify advanced fibrosis; it was not used to establish clinical decision thresholds or as a main conclusion, and the results still need to be validated in larger samples.

This study further observed changes in the proportion of significant liver fibrosis across different GGT levels. The proportions of ≥F2 significant liver fibrosis in the GGT < 100 U/L, 100 ≤ GGT ≤ 300 U/L, and GGT > 300 U/L groups were 19.0%, 56.3%, and 69.6%, respectively, showing a significant linear increasing trend (*χ*^2^ trend = 42.90, *P* < 0.001), suggesting a graded relationship between elevated GGT and increased proportion of histologically significant liver fibrosis. It should be noted that 100 U/L and 300 U/L are exploratory stratification cutoffs, set with reference to previous studies (16–19), while the 123.0 U/L obtained from ROC analysis is a data-driven optimal cutoff in this study; the two have different purposes. Because the literature basis for 100 U/L and 300 U/L partly comes from infantile cholestasis studies, and the median age of this cohort was 47 months, the applicability of these stratification cutoffs in this cohort still needs further validation. The optimal cutoff may be affected by age structure, disease spectrum, and biliary inflammatory status, and 123.0 U/L should not be used as a universal diagnostic cutoff for significant liver fibrosis in pediatric CDC. Therefore, this study is more suitable for using GGT level as an auxiliary clue suggesting the possibility of significant liver fibrosis, rather than a definitive diagnostic threshold.

This study has several limitations. First, it was a single-center retrospective study, and all included subjects underwent intraoperative liver biopsy, which may introduce selection bias and spectrum bias, and the results cannot be extrapolated to all children with CDC. Second, METAVIR is not specific for pediatric CDC, and biliary fibrosis may lead to staging misclassification; pathological staging may still have interobserver differences. Third, a history of preoperative cholangitis cannot determine whether cholangitis was active at the time of blood sampling, and residual confounding cannot be completely excluded. Fourth, GPR and APRI used a uniform ULN; sensitivity analysis using age/sex reference ranges did not substantially change the conclusions, but individualized ULN was lacking and still needs validation. Fifth, there was no systematic follow-up, so outcomes such as F3–F4, postoperative liver function recovery, recurrent cholangitis, reoperation, and fibrosis regression could not be evaluated; ≥F3 included only 32 cases and was exploratory. Sixth, the optimal cutoff was derived from the Youden index in the same cohort; Bootstrap only assessed stability and cannot replace external validation, so overfitting is possible. FIB-4 had an AUC close to 0.5, and its Bootstrap cutoff was unstable, so it was not clinically interpreted. PPV/NPV are affected by prevalence and should not be extrapolated. Finally, elastography was not routinely performed, and its accessibility in children is limited, so the performance of serum markers vs. elastography and their combined value could not be compared.

## 5 Conclusions

In this cohort, GGT showed relatively good discriminatory performance for significant liver fibrosis in children with CDC, but its AUC was not significantly superior to GPR, FCI, S-index, or APRI. GGT level was associated with histologically significant liver fibrosis and can serve as an auxiliary serum marker for preoperative assessment of liver fibrosis degree. However, given its influence by biliary inflammation and cholestasis, it still cannot replace histopathological evaluation, and further validation through multicenter studies is needed.

## Data Availability

The original contributions presented in the study are included in the article/Supplementary Material, further inquiries can be directed to the corresponding author.
