# Supplementary material for: Comparison of non-invasive serum markers for discriminating significant liver fibrosis in children with choledochal cyst
Source: Front Pediatr. 2026 Oct 27;14:1992446. doi: 10.3389/fped.2026.1992446 (PMC13748413; doi:10.3389/fped.2026.1992446)
Supplement: Supplementary file 1 [file Table1.docx]

## **Supplementary Table S1.** 2×2 classification counts and diagnostic performance at the optimal cutoff for significant fibrosis (≥F2)

| **Marker** | **Positive criterion** | **TP** | **FN** | **TN** | **FP** | **Sensitivity (95%CI)** | **Specificity (95%CI)** | **PPV (95%CI)** | **NPV (95%CI)** |
| --- | --- | --- | --- | --- | --- | --- | --- | --- | --- |
| GGT | ≥123.0 | 95 | 11 | 79 | 34 | 89.6% (82.2–94.7) | 69.9% (60.6–78.2) | 73.6% (65.2–81.0) | 87.8% (79.2–93.7) |
| GPR | ≥0.857 | 93 | 13 | 79 | 34 | 87.7% (79.9–93.3) | 69.9% (60.6–78.2) | 73.2% (64.7–80.6) | 85.9% (77.3–92.0) |
| AAR | ≤1.141 | 76 | 30 | 72 | 41 | 71.7% (62.1–80.0) | 63.7% (54.1–72.6) | 65.0% (55.6–73.6) | 70.6% (60.8–79.3) |
| APRI | ≥0.406 | 88 | 18 | 80 | 33 | 83.0% (74.5–89.6) | 70.8% (61.5–79.0) | 72.7% (63.9–80.4) | 81.6% (72.7–88.5) |
| FIB-4 | Not definedᵃ | 63 | 43 | 60 | 53 | 59.4% (49.5–68.9) | 53.1% (43.5–62.5) | 54.3% (44.8–63.6) | 58.3% (48.2–68.0) |
| FCI | ≥0.230 | 90 | 16 | 77 | 36 | 84.9% (76.8–91.0) | 68.1% (58.7–76.8) | 71.4% (62.7–79.0) | 82.8% (73.8–89.7) |
| S-index | ≥0.152 | 95 | 11 | 76 | 37 | 89.6% (82.2–94.7) | 67.3% (57.8–75.9) | 72.0% (63.5–79.4) | 87.4% (78.6–93.5) |

Note: ᵃThe AUC of FIB-4 was close to the random level (0.528), and its optimal cutoff based on the Youden index has no clinical interpretability; therefore, no positive criterion was defined. Its sensitivity, specificity, PPV, and NPV 95% CIs are for reference only. The 95% CIs were calculated using the Clopper-Pearson exact binomial method.

## **Supplementary Table S2**. Pairwise DeLong comparisons among seven serum markers for diagnosing significant liver fibrosis (≥F2)

| **Comparison** | **AUC difference** | **95% CI** | **P value** | **Holm-adjusted P value** |
| --- | --- | --- | --- | --- |
| GGT vs GPR | 0.012 | -0.004–0.028 | 0.153 | 1.000 |
| GGT vs AAR | 0.121 | 0.059–0.184 | <0.001 | 0.002 |
| GGT vs APRI | 0.055 | 0.003–0.108 | 0.039 | 0.430 |
| GGT vs FIB-4 | 0.298 | 0.203–0.394 | <0.001 | <0.001 |
| GGT vs FCI | 0.024 | -0.025–0.073 | 0.341 | 1.000 |
| GGT vs S-index | 0.017 | -0.002–0.037 | 0.081 | 0.812 |
| GPR vs AAR | 0.109 | 0.047–0.172 | <0.001 | 0.010 |
| GPR vs APRI | 0.043 | -0.007–0.094 | 0.093 | 0.834 |
| GPR vs FIB-4 | 0.286 | 0.193–0.379 | <0.001 | <0.001 |
| GPR vs FCI | 0.012 | -0.039–0.063 | 0.643 | 1.000 |
| GPR vs S-index | 0.005 | -0.007–0.018 | 0.409 | 1.000 |
| AAR vs APRI | -0.066 | -0.150–0.018 | 0.124 | 0.988 |
| AAR vs FIB-4 | 0.177 | 0.064–0.290 | 0.002 | 0.029 |
| AAR vs FCI | -0.097 | -0.175–-0.020 | 0.014 | 0.170 |
| AAR vs S-index | -0.104 | -0.167–-0.041 | 0.001 | 0.016 |
| APRI vs FIB-4 | 0.243 | 0.163–0.323 | <0.001 | <0.001 |
| APRI vs FCI | -0.031 | -0.087–0.024 | 0.266 | 1.000 |
| APRI vs S-index | -0.038 | -0.092–0.016 | 0.167 | 1.000 |
| FIB-4 vs FCI | -0.274 | -0.360–-0.188 | <0.001 | <0.001 |
| FIB-4 vs S-index | -0.281 | -0.376–-0.186 | <0.001 | <0.001 |
| FCI vs S-index | -0.007 | -0.058–0.045 | 0.806 | 1.000 |

## **Supplementary Table S3**. Bootstrap internal validation (2000 resamples)

| **Marker** | **Bootstrap AUC 95% CI** | **Bootstrap cutoff percentile range** |
| --- | --- | --- |
| GGT | 0.769–0.879 | 86–161 |
| GPR | 0.753–0.869 | 0.716–1.166 |
| AAR | 0.635–0.772 | 0.967–2.027 |
| APRI | 0.705–0.832 | 0.406–0.497 |
| FIB-4 | 0.453–0.606 | Not calculatedᵃ |
| FCI | 0.742–0.859 | 0.164–0.316 |
| S-index | 0.748–0.865 | 0.152–0.206 |

Note: ᵃThe AUC of FIB-4 was close to the random classification level (0.528), and the stability of its Bootstrap optimal cutoff was poor; therefore, no clinical threshold interpretation was performed.
